# Interdependent Networks: A Data Science Perspective

**DOI:** 10.1016/j.patter.2020.100003

**Published:** 2020-03-20

**Authors:** M. Hadi Amini, Ahmed Imteaj, Panos M. Pardalos

**Affiliations:** 1 School of Computing and Information Sciences, Florida International University, Miami, FL 33199, USA; 2 Sustainability, Optimization, and Learning for InterDependent Networks Laboratory (Solid Lab), Florida International University, Miami, FL 33199, USA; 3 Department of Industrial and Systems Engineering, University of Florida, Gainesville, FL 32611, USA

**Keywords:** interdependent networks, data science, interdependent decision making, multiplex networks, heterogeneity, societal network, large-scale optimization problem, transportation network, energy network, financial network, healthcare network, water network

## Abstract

Traditionally, networks have been studied in an independent fashion. With the emergence of novel smart city technologies, coupling among networks has been strengthened. To capture the ever-increasing coupling, we explain the notion of interdependent networks, i.e., multi-layered networks with shared decision-making entities, and shared sensing infrastructures with interdisciplinary applications. The main challenge is how to develop data analytics solutions that are capable of enabling interdependent decision making. One of the emerging solutions is agent-based distributed decision making among heterogeneous agents and entities when their decisions are affected by multiple networks. We first provide a big picture of real-world interdependent networks in the context of smart city infrastructures. We then provide an outline of potential challenges and solutions from a data science perspective. We discuss potential hindrances to ensure reliable communication among intelligent agents from different networks. We explore future research directions at the intersection of network science and data science.

## The Bigger Picture

This article provides a holistic overview of interdependent cyber-physical-societal networks. We envision the subsequent research directions that require contribution of the data science community as well as interdisciplinary collaboration with network scientists, social scientists, computer scientists, and engineers to tackle the emerging problems raised by the notion of interdependent networks: (1) developing novel algorithms for data analytics and enabling interdependent decision making, (2) proposing holistic models that are capable of capturing the interdependence among human-centered multi-layer critical infrastructures, and (3) developing efficient solutions that are capable of finding globally optimum solutions using information from each network as well as modeling the interdependent information exchange. In addition to these directions, we outline policy and access-control issues, including conflict of interest among stakeholders and operators of each network. Successful implementation and development of an interdependent data analytics framework and its required algorithms will improve the quality of life of citizens by enabling globally optimum decision making, increasing efficiency, preserving privacy of intelligent agents, and reducing operational cost of interdependent networks. Further reading: Sustainable Interdependent Networks book series (interdependentnetworks.com) and *Optimization, Learning, and Control for Interdependent Complex Networks* (edited by M.H. Amini).

## Graphical Abstract

**Figure:**
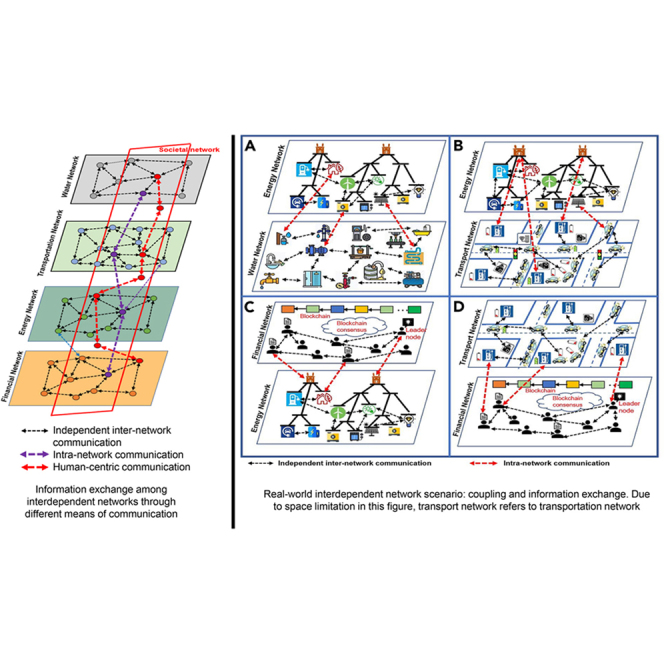

## Teaser

Real-world complex networks are coupled through shared entities and decision makers; e.g., there is a transition toward coupled smart cities infrastructures by integrating intelligent technologies. In this study, we provide a holistic overview of coupled societal-water-energy-economical-transportation (SWEET) networks as critical components of smart cities. We explore required methods to ensure optimal decision making and data analytics considering the increasing coupling of these human-centered networks. We finally provide future directions for researchers who work at the intersection of data science and network science.

## Main Text

### Introduction

Due to emerging coupling and interdependence among critical smart cities infrastructures,^1^^,^^2^ it is imperative to develop holistic data analytics and efficient decision-making techniques,^1^^,^^3^, ^4^, ^5^, ^6^, ^7^ such as agent-based distributed optimization algorithms^8^^,^^9^ and learning from distributed datasets.^10^ While interdependent networks and their underlying structure have been studied from the network science perspective,^11^, ^12^, ^13^, ^14^ which is crucial to enable an intuitive understanding of networks, there is a need to develop efficient data-driven algorithms to deal with decision-making problems in interdependent complex networks. Machine learning and data analytics lend themselves as promising solutions to deal with the complex nature of interdependent networks.^15^ Human-centered coupling is the pivot to the interaction among heterogeneous agents in interdependent networks. Critical infrastructures ultimately aim at serving society. Motivated by this ever-increasing human-centered interdependence, this Perspective article outlines the need for revisiting current data analytics approaches to capture this interdependence. Specifically, this paper investigates potential research directions at the intersection of network science and data science.

Baggio et al.^16^ have introduced three strategies based on economic and ecological changes of three communities in Arctic Alaska. Multiplex, undirected, and weighted network analysis were deployed to show that community robustness is affected by social changes more than resource depletion.^16^ A multi-slice framework is proposed by Much et al.^17^ to enable algorithmic detection of cohesive groups based on the generalized Laplacian dynamic method. This framework is efficiently applicable to time-dependent multiplex networks.^17^ Renoust et al.^18^ performed group cohesion analysis by developing a visual analytics system, referred to as Detangular. While the proposed method is based on the underlying structure of the network through dual linked views, the interdependence among the data of multiplex networks has not been considered.^18^ Kanawati^19^ conducted a brief survey of advances in multi-layer network mining. The role of multiplexity in networks has been investigated by Fu and Chen.^20^ Integrative analysis of heterogeneous omics data have been used by Wang et al.^21^ to develop an approach for identification of cancer subtypes. This approach analyzes each data category independently and integrates correlated patient data from different sources.^21^ Hence, interdependent decision making can extract meaningful relations and patterns by using interdependent networks data. Xu and Tian^22^ conducted a comprehensive survey of clustering algorithms. While the advantages and disadvantages, and the differences among clustering algorithms, are been discussed by these authors,^22^ interdependent decision making among the clusters is not considered.

The rest of this paper is organized as follows. We provide an overview of human-centered interdependent networks, followed by a comprehensive introduction to interdependent societal-water-energy-economical-transportation (SWEET) networks. We then provide more details of the networks within smart cities, explore interdependence among various networks under the umbrella of interdependent SWEET networks, and outline the future direction in data analytics for interdependent networks, followed by our conclusions.

### Interdependent Decision Making in Coupled Multi-layer Networks

Considering the interdependent information exchange among multi-layer networks, there is a crucial need to investigate these networks elaborately and obtain a holistic understanding of how they interact. Assuming that there are multiple layers of networks, e.g., water, transportation, energy, financial, healthcare, and societal networks, nodes within each layer can communicate with each other. Furthermore, all nodes within each network are dynamically interacting together as a cluster. Holtz et al.^23^ have explored the benefits of modeling societal networks to enhance clarifications and understanding of societal transitions. Particularly, they explained the advantage of modeling for large-scale societal systems and provided a direction to use modeling for variant transition studies. Fiksel^24^ discusses dynamic properties of societal network nodes, which fluctuate due to the influence of the adjacent nodes. He investigated societal networks for long-term evaluation for both deterministic and probabilistic transition rules. Furthermore, a dynamic modeling of disease outbreak is proposed by Eubank et al.,^25^ whereby they model the physical pattern generated from the movement of the individuals using a dynamic bipartite graph. The analyzed graph was generated using mobility data, land-use data, and census data. In this Perspective, our goal is to identify the probable issues that may arise while coupling several layers, making communication among them, and considering human-centered nodes for revealing informative knowledge. Specifically, we try to identify potential issues while enabling interdependent decision making in a network-of-networks environment, its underlying communication and information exchange requirements to make globally optimum decisions. To this end, we consider three major means of communication: (1) independent inter-network communication; (2) intra-network communication; and (3) human-centered communication. Some of the challenges to leverage these communication strategies are outlined below.

The first problem we may face for an interdependent network is that any node can be out of the current network range or the node becomes dead during communication. This can happen because of mobile/dynamic behavior of the nodes, topology of underlying network, scalability constraint, environmental factors, different attacks (e.g., sinkhole attack, jamming attack, DDoS attack, man-in-the-middle attack, or cyber-physical attacks), heterogeneous transmission range, power doom, or device bug. These factors result in dropping requests and violence while performing a transaction, and encounters both connection establishment delay and transmission delay of request and response during communication. Consequently, corresponding nodes that are trying to initiate communication and broadcast information require additional time to reestablish or reset the high-level state of the conversation.

Besides, during intra-communication of the networks, the conventional node by node-searching approach can lead to a high latency among origin and source agents. To mitigate this, an efficient node-traversing approach should be adapted to achieve a minimal delay.

Avoiding the refusal of communication by malicious nodes is another challenge in interdependent network communication. A node that is responsible for maintaining communication with a couple of other nodes (i.e., human-centered data-analytic) can act suspiciously by rejecting a request or dropping a transaction at the middle. The identification of such idiosyncratic behavior of the nodes is one of the major concerns in the communication network.

### Interdependent Societal-Water-Energy-Economical-Transportation Networks: A Comprehensive Holistic View of Critical Infrastructures

The concept of interdependent SWEET networks refers to the underlying smart cities infrastructures. Interdependence among these networks may cause cascading failure, i.e., a single node failure leads to cascading failure of other dependent nodes located in different networks. In such scenario, a small fraction of node failure causes huge fragmentation within the system. To tackle this issue, holistic modeling of network interdependency is crucial in data analytics for intelligent decision making and to design a more robust network of networks. Specifically, considering the exogenous data from interdependent networks while using data analytics for each layer improves the accuracy of the decisions for intelligent agents. It also facilitates data-driven identification of critical nodes that can contribute to mitigating loss due to sudden disruption within the connected partitions of the network. For instance, electrical power outage due to some catastrophe can be a relevant example, whereby a single failure of a microgrid can affect all the dependent nodes located in different networks. Buldyrev et al.^26^ suggested a framework for interdependent networks that helps us understand the interaction and robustness of nodes of different networks, presented a solution for preventing fragmentation due to sudden cascade, and analyzed how such interdependent network vulnerability is different from a single network failure. They mentioned that, when we fragment the networks, the nodes that belong to a giant fraction and connected to the finite portion of the overall network are functional, but the remaining nodes, which are separated and part of the small clusters, are nonfunctional. Thus, they considered only the giant clusters that are interconnected and excluded the small nonfunctional clusters. They presented an analytical solution by considering those functional nodes and analyzed how the removal of a critical fraction of nodes can lead to the failure of cascade nodes and create fragmentation of interdependent networks. They also discussed how the higher distribution of multi-networks can cause more random failure than a single network and highlighted how interdependent networks can be effective in building a robust network structure.^26^

### An Overview of Underlying Cyber/Physical/Societal Layers of Interdependent Networks

In this section, we first discuss core network entities that are available within the system, e.g., societal networks, healthcare networks, water networks, energy networks, and financial/economic networks. We highlight the coherence among these networks and explain how a network can benefit from interconnecting with other networks.

#### Societal Networks

Societal networks represent the concept of having access to multiple-layer networks while moving and sharing that information with other nodes within the community. For instance, a node residing in the water network can get access to water resources, and when it moves and wants to communicate with a node in the same network residing in the opposite edge, it can use the societal network node as intermediate node to reach the target node and hence establish communication. A similar concept can also be applied for the nodes located in two different networks, which is discussed in a later part of this subsection. Mucha et al.^17^ designed a framework regarding network quality functions that facilitate us to build a structure of random multi-slice layers, and their framework adapts the structure change with network variations considering time, multiplexity, and scalability. They mentioned that inter-slice network coupling has a different nature than those inter-slice networks that connect only with their neighboring slices. They also discussed the detection process of the community for multi-scale, time-dependent, and multiplex networks through varying coupling strength and community group members. Human-centered computing from a multimedia perspective is discussed James et al.^27^ and Aragon et al.,^28^ where the main properties of human-centered multimedia, their interactions, research agendas, and potential applications are analyzed. Besides, different issues regarding human-centered computing using a machine-learning approach is examined by Fiebrink and Gillies^29^ and Riedl.^30^ The theory of rumor spreading in complex social networks has been comprehensively explored by Nekovee et al.^31^

**Figure 1:**
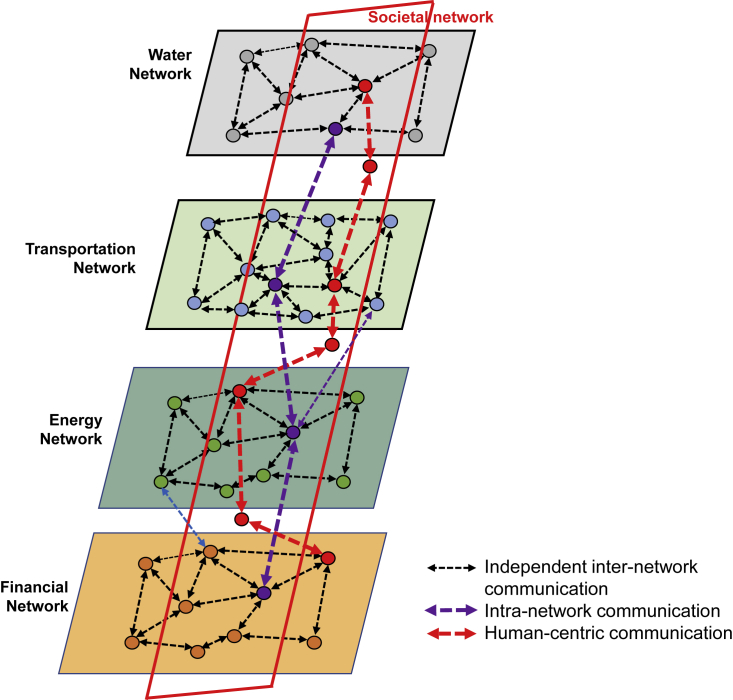
Human-Centered Cross-Layer Information Exchange in Interdependent Networks

In this Perspective, we consider societal networks as a common coupling network among all underlying interdependent networks. Societal network agents act as coupling nodes that facilitate communication from one network with another, which is potentially not connected and difficult to reach due to communication infrastructure constraints. In such a case, human-centered societal nodes can be effective, which will act as a bridge between two network agents and can contribute to successful interaction by taking some incentives. A societal network agent can also be considered as an entity that can carry information, make an intelligent route, establish communication, and disseminate knowledge among other interdependent networks. This kind of network is also useful when we have two different types of networks and each has its data policy, as well as permission access about information. In such a situation, a human-centered agent that may have common coupling with both of the networks can be beneficial for sharing information and carry out the transaction (Figure 1).^15^ An analysis of weighted and directed multiplex networks is presented by Baggio et al.,^16^ who considered ecological and economic changes of food flow in three small communities and explained how social relations have more impact on ensuring the robustness of community compared to available assets. They found that the interconnection of the community has more influence through the removal of significant social networks rather than vulnerabilities of resources. However, they did not consider the interdependence of societal networks and how social relations can captivate transportation or financial networks. For instance, two nodes that are socially connected and have random interaction are more likely to commit a transaction in financial networks. Besides, those two social network nodes can more often look for similar routes or transport to reach their target destination, or they can choose to purchase electricity from the same power network by being influenced through social interaction.

#### Healthcare Networks

**Figure 2:**
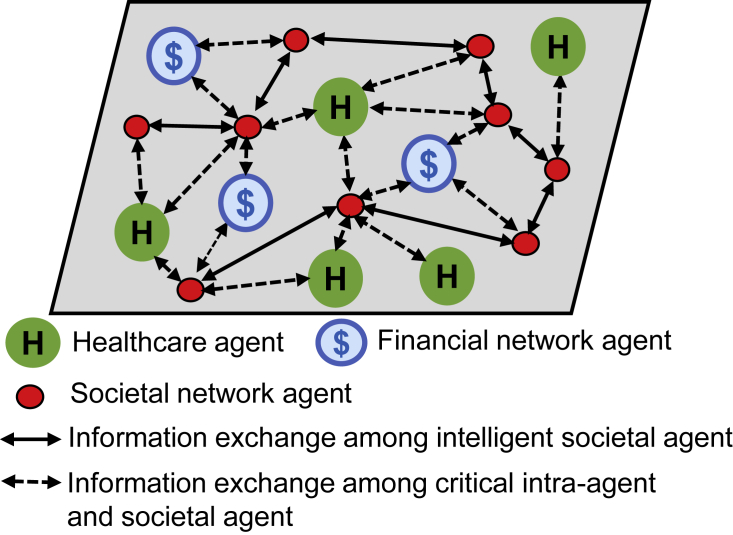
Heterogeneous Human-Centered Decision Making

Healthcare networks can be considered as the interconnection among the healthcare components to facilitate the patients with proper monitoring and services. This network can be operated more prudently if it can obtain better synchronization with other related networks that can contribute to the service continuation in a better way. For instance, a healthcare network can use the resource of energy networks when there is a lack of power supply to execute an operation. In such an emergency case, coupling with other networks can be crucial in terms of patient health and reputation of the service management. A high-level representation of human-centered decision making using healthcare and financial network agents is given in Figure 2. Transportation networks can play a vital role by providing necessary information about the shortest route, nearby gas station, or the current location of an ambulance. Integrating such a network with healthcare systems can help us to accelerate the throughput, cope with variant situations within an environment, and ensure consistency of service. Moreover, it can also interact with the financial network to make a transaction between the service management and patient that can help a patient to complete a transaction more securely and smoothly. Studies on cancer detection for interdependent tumor interaction,^32^ interdependent signals,^33^ and how different physical parameters (e.g., psychological and physical distress, pain and stress) are interdependent in cancer patients^34^, ^35^, ^36^ have been conducted. Such studies show that interdependency can tailor significant knowledge, which can help us to understand the detection of cancer more rigorously. We can apply a similar concept in terms of different networks, whereby a single independent network can only lead us to define some specific knowledge, whereas while coupling with some other interdependent networks they cumulatively can reveal more information and provide better service and facilities.

#### Water Networks

In the water network, the operation of supplying water among the nodes is dependent on the availability of electricity; thus, the distribution can be dependent on the power network and it is more variable. In such a distribution, the pumps used to supply water can also be used as energy reservoirs and hence can contribute to supplying electricity from the power network. Such types of coupling strategy of multi-networks can be beneficial to reveal hidden interconnections and ensure the robustness of system performance. We can formulate a more optimized function by considering those dependencies and can understand how multi-layer communication can lead us to achieve maximal benefit from the system. An optimal water flow (OWF) approach is presented by Singh and Kekatos,^37^ which incorporates the water pressure and flow constraints for a fixed speed of the water pump, pipes, reservoirs, and tanks. They relaxed the hydraulic constraint for the second-order cone and appended a penalty term for restoring the feasibility. By setting the proper weight to the penalty factor, a small originality gap is attained that gives OWF solutions. If we consider the water network without any other network dependency, the nodes can only fulfill their demand by using the same network nodes. However, if we introduce the concept of multiplex communication, the nodes within this network can derive benefit from another network by using their resources. The nodes that have enough power in the energy network can contribute to supply power in the water network by taking incentives. This will eliminate the waiting cost of the network when there is an outage of the main power station or node failure within the network.

#### Energy Networks

Energy networks are essential to enable continuous operation of critical infrastructures. There is an increasing number of sensors and measurement devices at various energy networks to collect and process network data and ensure reliable energy delivery. There is a wide range of studies concerning optimal operation of gas networks^38^ and power networks.^39^ While the collected data directly correspond to energy networks, they will affect the underlying electrified networks, e.g., water pumps in the water network, traffic lights in transportation networks, health monitoring equipment in healthcare, and computation centers in financial networks. Furthermore, as electricity customers are intelligent decision makers in societal networks, there is a strong coupling between power and societal networks. For instance, demand side management, including response programs^40^, ^41^, ^42^ and load management strategies,^43^^,^^44^ increase engagement of electricity consumers in the power market by allowing for customer participation in ensuring load-generation balance. Hence, a holistic understanding of energy networks and developing efficient data analytics to identify anomalies in these networks paves the way for the reliable and secure operation of electrified networks. While there is a rich literature on power network resilience,^45^ developing data analytics solutions that capture interdependence among power networks and societal networks is crucial in achieving a holistic understanding of these networks.

#### Economic/Financial Networks

In multi-layer peer-to-peer networks, customer privacy violence is a major concern as there is always a high chance of security breakdown and data leakage. To ensure security and maintain privacy of data, blockchain-based peer-to-peer networks^46^ can be a good solution. However, adopting blockchain to achieve economic/financial coupling for multi-layer networks may face some issues while performing transactions among different layers. In case of intra-communication blockchain networks, a transaction can be inserted within a block by attaining consensus from the mining nodes. However, a problem arises when the number of mining nodes within the network becomes very few and most of them are malicious. In such a case, there is a possibility of inserting improper transactions within the block due to consensus manipulation. Furthermore, if we consider communication among multi-layer networks and take into account that each layer has its blockchain network, we need to deal with various consensus mechanisms, variant data format, dissimilar blockchain size, or heterogeneous blockchain network nodes with different transmission capabilities.^47^, ^48^, ^49^ Furthermore, when a node tries to search for particular data, discovering probable blockchain and selection of the best suitable blockchain networks to communicate is another research problem. In such a case, we may have a performance-accuracy trade-off, i.e., a blockchain may have high throughput but low performance while another blockchain may have low throughput but high performance. In addition, when an interruption occurs during asset exchange among multiple nodes, switching time between different blockchains is a challenge. Also, directly switching to other chain may encounter an accessibility issue. Due to privacy, a blockchain network may fail to retrieve data directly from a chain, but may be accessible by routing through a neighboring blockchain. Additionally, due to mobility, a blockchain network may have very few nodes at a particular time, and if another blockchain network tries to make communication with a small-sized blockchain network, there is a high chance of obtaining wrong data due to 51% attack,^50^ selfish mining,^51^ or block withholding attack.^52^

### Interdependent Information Exchange in Critical Infrastructure

**Figure 3:**
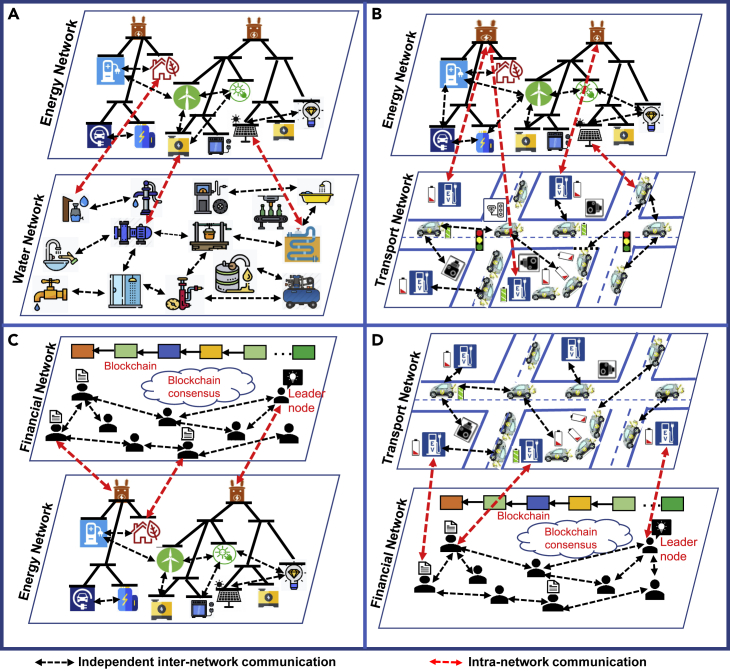
Examples of Real-World Interdependent Networks: Coupling and Information Exchange of Inter-/Intra-Networks Due to space limitation in this figure, “transport network” refers to “transportation network.”

Currently, the data for each network is stored in silos, e.g., healthcare nodes may not have interaction with the entity that has access to financial data or the transport data do not have access to energy data. For this reason, resources cannot be utilized in an optimized manner and different interesting knowledge remains unrevealed. To ensure optimal utilization of resources and discover informative knowledge, the coupling of multiple networks can be considered, whereby independent inter-network and intra-network communication can be made. Through inter-network communication, the same network entities can share resources and talk among themselves and in intra-network communication, and different network node entities can speak with each other about node and resource information, exchange data, and can provide support to attain resilience within the system. Hamich et al.^53^ and Kahrl and Roland-Holst^54^ combined water and energy data to examine the linkages between these two types of data to achieve sustainable resource management. Figure 3A shows the coupling and interactions of energy and water networks. For instance, if a node within the water network requires energy at any time instantly to continue its normal water supply operation, alternative power supply requests can be made to the energy network. The requested energy network can check out its resources and upon the availability of the requested resource and respond to a water network for further execution. Besides, if due to natural calamity, a power outage occurs in a region and water for power supply is interrupted, in such a case, energy from other networks can be provided for maintaining the continuation of water distribution. In Figure 3B, coupling among energy and transportation networks is illustrated, whereby these networks are interdependently operating to reach optimal decisions. The entity of the transportation network (e.g., vehicles, gas stations, and surveillance components) directly relies on the energy systems, and the discontinuation of such energy leads to malfunction of transportation networks. Various research groups^9^^,^^55^, ^56^, ^57^, ^58^ have explored the interdependent nature of power system and electrified transportation networks and have proposed frameworks and algorithms to make optimal decisions in both optimal power flow problems in power systems and optimal routing problems of electric vehicles. To address the interdependent planning of these two networks, Amini et al.^58^ proposed a simultaneous strategy for planning of distributed renewable generation and electric vehicle charging stations. Financial and energy networks are correlated in many aspects. For instance, to purchase energy from a power source, it may be necessary to communicate with the financial network for obtaining a reliable exchange of transaction, e.g., blockchain. A node that has permission to commit the transaction and push it to the block of the financial blockchain network may have access to utilizing energy from the energy network. In such a situation, a coupling of two such networks can be beneficial for the node entities to gain more resources at a certain time interval (Figure 3C). Siano et al.^59^ and Aitzhan and Svetinovic^60^ explained how blockchain-based transaction can be applied in the energy stock market for exchanging or sharing energy among the peer-to-peer communication nodes. They discussed how blockchain-based transactions can be adapted in the energy market for securing asset transfer and proper resource utilization. A group of leader nodes within the blockchain network can handle all the transaction requests from other networks and can propagate the requester information within the network channel. Thereafter, all the interested nodes can give a response to the leader request and, upon giving consent to the contract, can commit transactions to insert within the chain. The mining nodes can verify the transaction and reach a consensus to insert that transaction into the blockchain. In this way, the financial network nodes can be used in the completion of a secure transaction with the energy network, and this approach can be valuable when a node or a region faces power disturbance. Similarly, financial and transport networks can be sliced together to leverage transport and financial network functionalities to utilize each other's facilities (Figure 3D).

**Figure 4:**
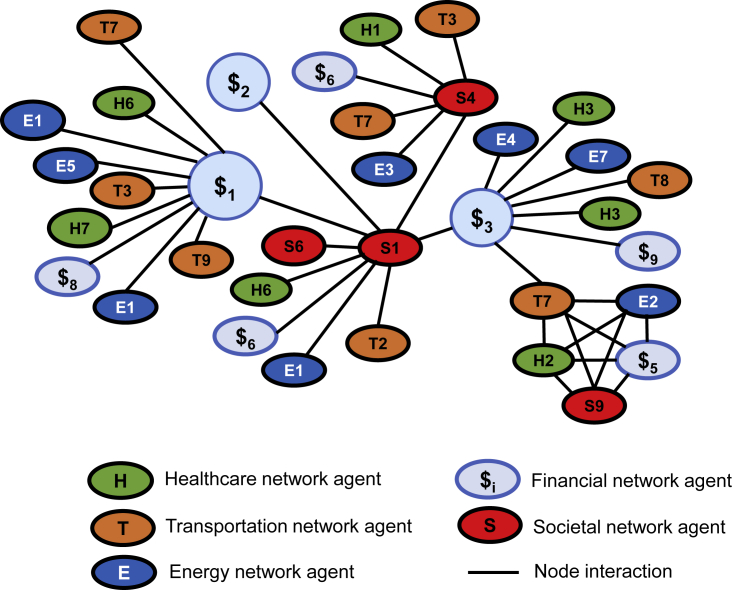
Interaction Map of Heterogeneous Agents from Societal, Healthcare, Transportation, Energy, and Financial Networks

We can track information exchange among nodes during a given period to monitor their interactions and regularity of successful communication. Using such interactive structures (Figure 4), we can quantify the node's availability or trust value, or predict interest in communicating with similar nodes later.

### Future Directions in Data Analytics for Interdependent Networks: Enabling Interdependent Decision Making

Based on the thorough investigation of cyber-physical-societal networks in this Perspective, we envision the following research directions that require the contribution of the data science community, as well as interdisciplinary collaboration with social scientists, computer scientists, and engineers, to tackle the emerging problems raised by the notion of interdependent networks:

• Developing novel algorithms for data analytics and enabling interdependent decision making
• Proposing holistic models that are capable of capturing the interdependence among human-centered multi-layer critical infrastructures
• Developing efficient solutions that are capable of finding globally optimum solutions using information from each network as well as modeling the interdependent information exchange

In addition to the aforementioned directions, we outline policy and access control issues, including conflict of interest among stakeholders and operators of each network. For instance, for an electric vehicle driver who receives information from the transportation network, energy system, and also financial network, how can we develop a holistic data analytics algorithm that takes into account these cross-layer data to enable actionable intelligence? Besides, if we consider coupling of the healthcare network with other networks (e.g., energy and transportation), then how can we discover a relationship between a patient who is using the healthcare network and the other related networks being used by the patient to obtain better service, or discover meaningful patterns while respecting the privacy of agents?

### Conclusions

In this Perspective, we explored potential directions for interdependent data analytics in human-centered multi-layer networks. We described how an entity can be beneficial in obtaining service through interdependent inter-communication and intra-communication asset sharing. Furthermore, using the concepts of human-centered network communication and coupling among different networks, we provided a pathway toward optimal resource utilization and explained how it can be applied in the presence of human agents. Such interactions can be performed when there is either more resource availability than demand, or resource shortage within a network. Taking into account different communication environment scenarios, we investigated potential challenges that an agent may face while dealing with different communication infrastructures, leveraging multi-modality data from various networks, or making efficient use of the shared resources. While the current data science solution provides efficient network-centered data analysis, we continue to explore an ever-increasing need to revisit these solutions, take into account human-centered factors, and model the interdependent decision-making infrastructure. Different kinds of emerging computing and information science problems (e.g., large-scale machine learning, big data analytics) and engineering problems (e.g., home automation, smart city, smart agriculture, large-scale product manufacturing) require tailored data-analytic algorithms to model and integrate our discussed interdependent networks notion. By adapting our multi-layer communication strategy through available communication means, network resources can be exploited and interdependent data sharing can ultimately benefit optimal decision making of all networks.
